# Supplementary material for: A framework for objectively comparing competing invasion percolation models based on highly-resolved image data
Source: PLoS One. 2026 Mar 23;21(3):e0327414. doi: 10.1371/journal.pone.0327414 (PMC13008257; doi:10.1371/journal.pone.0327414)
Supplement: S1 Table — (PDF) [file pone.0327414.s001.pdf]

Table 1: Table containing gas saturation values corresponding to the maximum metric value, Jaccard coefficient (J), Diffused Jaccard coefficient (low) (J_d^{low}), Diffused Jaccard coefficient (med) (J_d^{med}), and Diffused Jaccard coefficient (high) (J_d^{high}).

	**Injection rate**	**10 ml/min**				**100ml/min**				**250ml/min**				
	**Models**	**1**	**2**	**3**	**4**	**1**	**2**	**3**	**4**	**1**	**2**	**3**	**4**	
**Triplicate Experiments**	**A**	0.28	0.28	0.20	0.36	0.28	0.36	0.36	0.44	0.12	0.12	0.12	0.28	J
	**B**	0.28	0.28	0.36	0.44	0.36	0.36	0.44	0.28	0.12	0.12	0.12	0.28	
	**C**	0.28	0.28	0.36	0.44	0.36	0.36	0.44	0.28	0.28	0.28	0.12	0.28	
	**A**	0.44	0.02[a]	0.20	0.28	0.28	0.28	0.44	0.44	0.02[a]	0.02[a]	0.44	0.28	J_d^{low}
	**B**	0.02[a]	0.02[a]	0.36	0.44	0.36	0.36	0.44	0.44	0.28	0.28	0.28	0.20	
	**C**	0.20	0.20	0.36	0.20	0.28	0.28	0.44	0.02[a]	0.28	0.28	0.28[b]	0.28	
	**A**	0.44	0.20	0.20	0.20	0.28	0.28	0.44	0.02[a]	0.02[a]	0.02[a]	0.20	0.02[a]	J_d^{med}
	**B**	0.20	0.20	0.36	0.12	0.28	0.28	0.44	0.44	0.02[a]	0.02[a]	0.12	0.12	
	**C**	0.20	0.20	0.20	0.44	0.20	0.20	0.44	0.36	0.02[a]	0.02[a]	0.02[a]	0.02[a]	
	**A**	0.12	0.36	0.20	0.20	0.44	0.02[a]	0.44	0.44	0.12	0.12	0.36	0.12	J_d^{high}
	**B**	0.44	0.36	0.12	0.20	0.12	0.12	0.44	0.44	0.20	0.02[a]	0.12[c]	0.12	
	**C**	0.36	0.20	0.28	0.02[a]	0.36	0.02[a]	0.44	0.02[a]	0.36	0.12	0.02[a]	0.12	

[a] Same metric values also obtained for a gas saturation value of 0.04

[b] Same metric values also obtained for a gas saturation value of 0.36

[c] Same metric values also obtained for a gas saturation value of 0.20
