# Supplementary material for: A framework for objectively comparing competing invasion percolation models based on highly-resolved image data
Source: PLoS One. 2026 Mar 23;21(3):e0327414. doi: 10.1371/journal.pone.0327414 (PMC13008257; doi:10.1371/journal.pone.0327414)
Supplement: S3 Fig — (PDF) [file pone.0327414.s003.pdf]

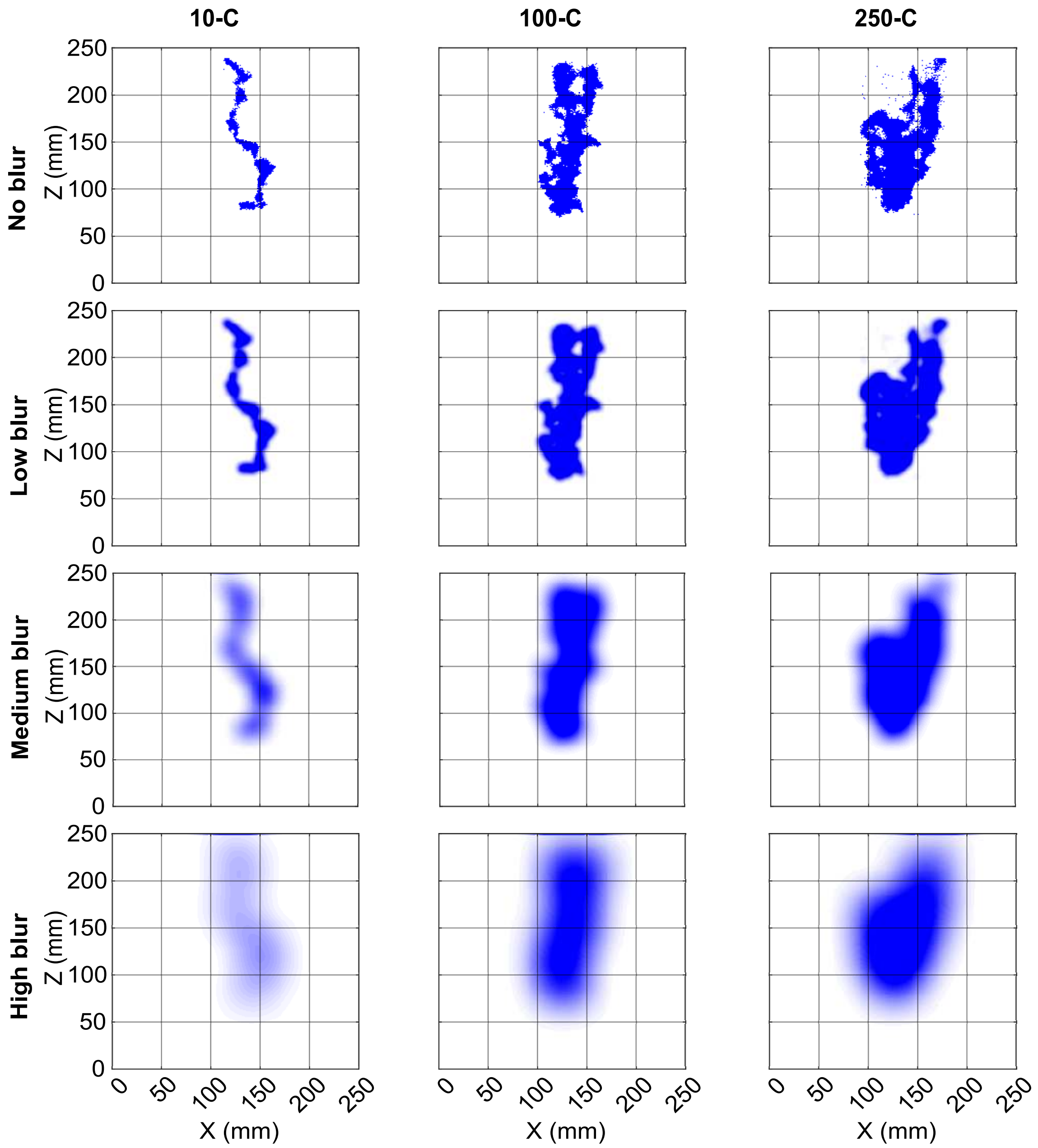

Figure S3: Final experimental image of the experiments 10-C, 100-C and 250-C. Row 2-4 contains the blurred version of the images of Row 1 for the three different blur-radii.
