# Supplementary material for: A framework for objectively comparing competing invasion percolation models based on highly-resolved image data
Source: PLoS One. 2026 Mar 23;21(3):e0327414. doi: 10.1371/journal.pone.0327414 (PMC13008257; doi:10.1371/journal.pone.0327414)
Supplement: S11 Fig — (PDF) [file pone.0327414.s011.pdf]

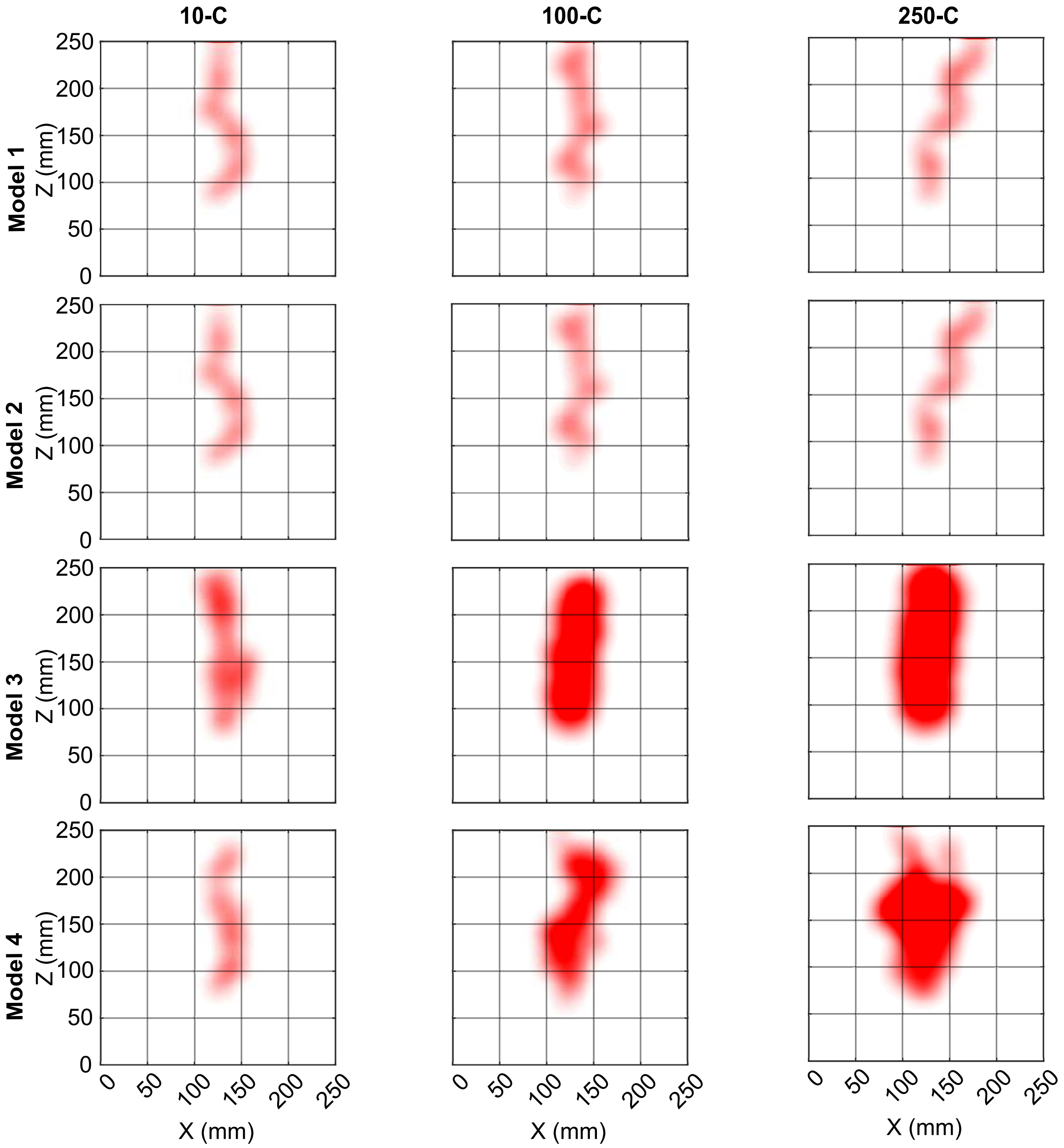

Figure S11: Model images for the different model versions with the best fit to blurred experimental images (with maximum Diffused Jaccard (med) value) from experiment no. 10-C, 100-C and 250-C. Row 1, Row 2, Row 3 and Row 4 correspond to Model 1, Model 2, Model 3 and Model 4, respectively.
