# Supplementary material for: A framework for objectively comparing competing invasion percolation models based on highly-resolved image data
Source: PLoS One. 2026 Mar 23;21(3):e0327414. doi: 10.1371/journal.pone.0327414 (PMC13008257; doi:10.1371/journal.pone.0327414)
Supplement: S15 Appendix — (PDF) [file pone.0327414.s015.pdf]

Appendix: Influence of inertial forces for the experiments of this study

Here, we examine how inertial forces influence the experimental conditions in our study. We conceptualize the injected gas as forming an initial spherical interface with a radius set by the injection needle. Our aim is to determine the radius of this expanding gas sphere beyond which inertial effects become small compared with viscous and capillary forces. To do this, we evaluate the Reynolds number (Re, ratio of inertial to viscous forces) and the Weber number (We, ratio of inertial to capillary forces):

$$Re = \frac{\rho_g Q r}{A \mu_g} \tag{1}$$

$$We = \frac{\rho_g Q^2 r}{A^2 \sigma} \tag{2}$$

where, ρ_g and μ_g are the density and dynamic viscosity of gas (air), Q is the gas-injection rate, r is the radius of the gas sphere, A is the interfacial area of the sphere, and σ is the air-water interfacial tension.

We rewrite Equation 1 and Equation 2 for the radius r of the expanding gas sphere, and for $Re = 1$ and $We = 1$.

$$r(Re = 1) = \frac{\rho_g Q}{4\pi \mu_g} \tag{3}$$

$$r(We = 1) = \left(\frac{\rho_g Q^2}{16\pi^2 \sigma} \right)^{1/3} \tag{4}$$

Using the values of $\rho_g = 3$ [kg/m^3], $\mu_g = 0.019 \times 10^{-3}$ [N/m^{2}s], and $\sigma = 0.071$ [N/m] we obtain $r(Re = 1)$ and $r(We = 1)$ as follows for the different experiments:

Table 1: Critical radius at which inertial forces become negligible relative to viscous forces (Re = 1) and capillary forces (We = 1) for different gas injection rates.

**Injection rate [ml/min]**	$r(\mathrm{Re} = 1)$ **[mm]**	$r(\mathrm{We} = 1)$ **[mm]**
10	2.09	0.02
100	20.9	0.07
250	52.4	0.13

We observe that even for the highest injection rate (250 ml/min), inertial forces exceed capillary forces only within a region smaller than 0.2 mm from the gas-injection point, which is less than the mean grain diameter of the sand ($d50 = 0.713 \pm 0.023$). This indicates that, at both pore and continuum scales, surface tension dominates over gas inertia. At the same injection rate, inertial forces exceed viscous forces up to roughly 50 mm from the inlet, but this range is still well below the size of the sandbox (125 mm to each side), and capillary forces remain the controlling factor throughout. Overall, these estimates show that inertial forces do not determine the displacement patterns observed in our experiments; capillary and viscous forces do.
