# Supplementary material for: A framework for objectively comparing competing invasion percolation models based on highly-resolved image data
Source: PLoS One. 2026 Mar 23;21(3):e0327414. doi: 10.1371/journal.pone.0327414 (PMC13008257; doi:10.1371/journal.pone.0327414)
Supplement: Attachment — Submitted filename: ResponseLetter_final.docx [file pone.0327414.s016.docx]

# Response to Editor and Reviewers

Dear Prof. Reuben O'Dea,

We would like to sincerely thank you and the reviewers for your thoughtful and constructive comments on our manuscript : PONE-D-25-31748: “A framework for objectively comparing competing invasion percolation models based on highly-resolved image data”. We have carefully considered all the feedback and revised the manuscript accordingly. Below, we provide detailed responses to each comment, along with a summary of the changes made in the revised version.

We believe these revisions have significantly improved the quality and clarity of our manuscript.

## General Comments

We have made the following general improvements to the manuscript based on the reviewers’ feedback:

## Journal Requirements:

1. When submitting your revision, we need you to address these additional requirements.

Please ensure that your manuscript meets PLOS ONE's style requirements, including those for file naming. The PLOS ONE style templates can be found at

https://journals.plos.org/plosone/s/file?id=wjVg/PLOSOne_formatting_sample_main_body.pdf and

<https://journals.plos.org/plosone/s/file?id=ba62/PLOSOne_formatting_sample_title_authors_affiliations.pdf>

Thank you for the comment. This has been duly noted.

2. Thank you for stating the following financial disclosure:

German Research Foundation (DFG) for financial support of this project within the Research Training Group GRK1829 “Integrated  Hydrosystem  Modelling” and the Cluster of Excellence EXC 2075 “Data-integrated Simulation Science (SimTech)” at the University of Stuttgart under Germany’s Excellence Strategy - EXC 2075 - 39074001

Please state what role the funders took in the study.  If the funders had no role, please state: "The funders had no role in study design, data collection and analysis, decision to publish, or preparation of the manuscript."

If this statement is not correct you must amend it as needed.

Please include this amended Role of Funder statement in your cover letter; we will change the online submission form on your behalf.

The funders had no role in study design, data collection and analysis, decision to publish, or preparation of the manuscript.

3. Thank you for stating the following in the Acknowledgments Section of your manuscript:

The authors would like to thank the German Research Foundation (DFG) for financial 851 support of this project within the Research Training Group GRK1829 “Integrated 852 Hydrosystem Modelling” and the Cluster of Excellence EXC 2075 “Data-integrated 853 Simulation Science (SimTech)” at the University of Stuttgart under Germany’s 854 Excellence Strategy - EXC 2075 - 39074001. The authors would also like to thank Dr 855 Luciana Chavez Rodriguez from the Wageningen University and Research, 856 Netherlands for her constructive feedback on an early version of this manuscript.

We note that you have provided funding information that is not currently declared in your Funding Statement. However, funding information should not appear in the Acknowledgments section or other areas of your manuscript. We will only publish funding information present in the Funding Statement section of the online submission form.

Please remove any funding-related text from the manuscript and let us know how you would like to update your Funding Statement. Currently, your Funding Statement reads as follows:

German Research Foundation (DFG) for financial support of this project within the Research Training Group GRK1829 “Integrated  Hydrosystem  Modelling” and the Cluster of Excellence EXC 2075 “Data-integrated Simulation Science (SimTech)” at the University of Stuttgart under Germany’s Excellence Strategy - EXC 2075 - 39074001

Please include your amended statements within your cover letter; we will change the online submission form on your behalf.

Ans:

Thank you for the information. The acknowledgement section has been modified. Our funding statement should be as follows:

“The authors would like to thank the German Research Foundation (DFG) for financial support of this project within the Research Training Group GRK1829 “Integrated Hydrosystem Modelling” and the Cluster of Excellence EXC 2075 “Data-integrated Simulation Science (SimTech)” at the University of Stuttgart under Germany’s Excellence Strategy - EXC 2075 - 39074001.”

4. Thank you for uploading your study's underlying data set. Unfortunately, the repository you have noted in your Data Availability statement does not qualify as an acceptable data repository according to PLOS's standards.

At this time, please upload the minimal data set necessary to replicate your study's findings to a stable, public repository (such as figshare or Dryad) and provide us with the relevant URLs, DOIs, or accession numbers that may be used to access these data. For a list of recommended repositories and additional information on PLOS standards for data deposition, please see <https://journals.plos.org/plosone/s/recommended-repositories>.

Ans: As per the correspondence with the editor, the repositories would be made public upon acceptance.

5. When completing the data availability statement of the submission form, you indicated that you will make your data available on acceptance. We strongly recommend all authors decide on a data sharing plan before acceptance, as the process can be lengthy and hold up publication timelines. Please note that, though access restrictions are acceptable now, your entire data will need to be made freely accessible if your manuscript is accepted for publication. This policy applies to all data except where public deposition would breach compliance with the protocol approved by your research ethics board. If you are unable to adhere to our open data policy, please kindly revise your statement to explain your reasoning and we will seek the editor's input on an exemption. Please be assured that, once you have provided your new statement, the assessment of your exemption will not hold up the peer review process.

Ans:

The concern has been duly noted. The repository will be made public as soon as the paper gets accepted. The background processes for the same have been dealt with.

6. Please amend either the abstract on the online submission form (via Edit Submission) or the abstract in the manuscript so that they are identical.

Ans:

We have ensured that the Abstracts are identical.

7. Please upload a copy of S14 Figure, to which you refer in your text on page 22. If the figure is no longer to be included as part of the submission, please remove all reference to it within the text.

Ans:

Thank you for observing the missing figure in the Supporting information. The S14 Figure is now uploaded.

## Response to Reviewer #1

**Reviewer #1:** This study compares four macroscopic Invasion Percolation (IP) models for simulating gas flow in water-saturated porous media beyond the discontinuous regime. Using light transmission imaging and randomised entry pressure fields, the models are tested against experiments in transitional and continuous flow regimes. A diffused Jaccard coefficient is used to evaluate performance. Results show that some IP models are effective outside their traditional application range, and that initial entry pressure fields have a significant influence on outcomes. The study presents a generalizable framework for model evaluation using high-resolution spatial and temporal data. A minor revision is recommended to improve clarity and quality.

We would like to thank you for your time and feedback on our manuscript. Please find the answers to the individual questions raised below.

1. Introduction:
In Lines 74-75, as for the discontinuous flow regime, discrete gas bubbles could be modelled by coupling buoyancy-driven bubbly flow in porous medium with Navier-Stokes equations rather than capillary-viscous-inertia-driven air slugs and air continuous flow. This model has been well-developed in COMOSAL.
Recommended work: Ma, Y., Yan, G., & Scheuermann, A. (2022). Discrete bubble flow in granular porous media via multiphase computational fluid dynamic simulation. Frontiers in Earth Science, 10, 947625.

Ans:

We thank the reviewer for suggesting this reference. We have read the recommended paper carefully and found that its modelling approach is indeed relevant to our study. Accordingly, we have incorporated this technique into the revised manuscript in lines 101-103.

“Modified continuum models formulated at the pore scale—using the Navier–Stokes equations to represent bubbly gas transport—have also been used to simulate discontinuous gas flow; however, this approach also has high computational cost, thus limiting their practical applicability at larger scales [75].”

2. Methodology:
Figure 1 is a good illustration of IP, while you forgot to provide a photo of your IP model in both the main text and the appendix. It would be beneficial to draw the dimensions of the IP model, the initial and boundary conditions applied to it, and include a scale bar on the IP images.
Additionally, the optical calibration between physical length and pixel should be provided, along with the imaging methods (camera, resolution, frame per second, etc.), image processing (grayscale threshold for binarization, noise filter algorithm, etc.), and image analysis. Not all details are necessary, but some key information should be mentioned for experimental replication.
Line 452: No indentation ahead of notation starting with “where”. Please fixe the same issues elsewhere.

Ans:

We thank the reviewer for raising this point. The intention of the remark is not entirely clear to us, so we address it under two possible interpretations below.

(A) If the reviewer is referring to the computational models (Invasion Percolation models) used in the study, our response is as follows:

We would like to clarify that the Invasion-Percolation (IP) models employed in our model comparison framework in this study have been well established and thoroughly described in the cited literature. Also, we have included all the relevant information on the general background of these models in Lines 124- 181. For the 4 specific models we have further included the description with equations in Sections 2.2-2.5. The model image outputs in the figure, are from these four corresponding models. All input parameter values used in the models are provided in Table 1.

The domain size of both the experiments and the models was kept consistent at 250 mm × 250 mm, corresponding to the size of the output images from the models. These dimensions are maintained in the figures of the manuscript; hence, a scale bar has not been included.

Because the primary aim of this work is to develop and demonstrate the evaluation framework—rather than to elaborate on the experimental setup or modelling procedures—we refer readers to the original publication for full technical details needed for replication.

(B) Alternatively, if the reviewer is referring to the experiments, then our response is as follows:

We have added the following lines to the revised manuscript in line numbers 282-294:

“In this method, the back of the cell is lit, and digital images or videos of gas injection are recorded. In the experiments used in this study, imaging was performed using a high-resolution camera (Canon EOS 6D equipped with a Canon EF 17–40 mm lens). The videos were captured at a resolution of 1920 × 1080 pixels and a frame rate of 29.97 frames s−1 for the duration of each experiment. The recordings were subsequently processed to extract still-frame images, which were cropped to include only the face of the cell and then converted to grayscale intensity fields. Individual pixel intensity values of these raw images are averaged over a block size of 1 × 1 mm, and the intensity values of the block are used to calculate the optical density (OD) [42] values. A median filter is applied to a 3 × 3 pixel (0.75 × 0.75 mm) neighbourhood so that the resulting OD field reflects variations over areas comparable to, but not smaller than, the diameter of an individual grain.”

The experimental data processing methodology is described in detail by Van de Ven et al. (2019). In this manuscript, we provide only the information necessary to support the evaluation framework presented here, but now adding a few clarifying details as recommended by the reviewer.

Because the primary aim of this work is to develop and demonstrate the evaluation framework—rather than to elaborate on the experimental setup or modelling procedures—we refer readers to the original publication for full technical details needed for replication.

The indentation issues noted by the reviewer have been carefully corrected in the revised manuscript.

3. Results and discussions:
Have you paid attention to the high gas injection-induced inertia effect in two immiscible phase seepage flow in porous media, as high injection flow rate may trigger dynamic nonequilibrium capillary water flow. These could be verified by comparing the Reynolds number and the Weber number to assess the relative contributions of inertial, viscous, and capillary forces.
Recommended publication: Yan, G., Li, Z., Bore, T., Torres, S. A. G., Scheuermann, A., & Li, L. (2022). A lattice Boltzmann exploration of two-phase displacement in 2D porous media under various pressure boundary conditions. Journal of Rock Mechanics and Geotechnical Engineering, 14(6), 1782-1798.

Ans:

We thank the reviewer for this comment. In determining the Reynolds and Weber numbers for our experiments, we drew on the “near-source” and “far-source” conceptual framework for gas injection into water-saturated porous media introduced by Selker et al. (2006). This framework guided our use of the inflating-sphere approximation to estimate the radii at which inertial, viscous, and capillary forces become dominant. We have added a brief explanation of this approach in S15 Appendix of the revised manuscript.

“Here, we examine how inertial forces influence the experimental conditions in our study. We conceptualize the injected gas as forming an initial spherical interface with a radius set by the injection needle. Our aim is to determine the radius of this expanding gas sphere beyond which inertial effects become small compared with viscous and capillary forces. To do this, we evaluate the Reynolds number (Re, ratio of inertial to viscous forces) and the Weber number (We, ratio of inertial to capillary forces):

Re = (ρ_g Q r) / (A μ_g)
We = (ρ_g Q^2 r) / (A^2 σ)

where ρ_g and μ_g are the density and dynamic viscosity of gas (air), Q is the gas-injection rate, r is the radius of the gas sphere, A is the interfacial area of the sphere, and σ is the air–water interfacial tension.

We rewrite the equations for the radius *r* of the expanding gas sphere for the cases Re = 1 and We = 1:

r(Re = 1) = (ρ_g Q) / (4 π μ_g)

r(We = 1) = ( (ρ_g Q^2) / (16 π^2 σ) )^(1/3)

Using the values ρ_g = 3 kg/m³, μ_g = 0.019 × 10⁻³ N·s/m², and σ = 0.071 N/m, we obtain the following results for the different experiments:

| **Injection rate (ml/min)** | **r (Re = 1) [mm]** | **r (We = 1) [mm]** |
| --- | --- | --- |
| 10 | 2.09 | 0.02 |
| 100 | 20.9 | 0.07 |
| 250 | 52.4 | 0.13 |

We observe that even for the highest injection rate (250 ml/min), inertial forces exceed capillary forces only within a region smaller than 0.2 mm from the gas-injection point, which is less than the mean grain diameter of the sand (d₅₀ = 0.713 ± 0.023 mm). This indicates that, at both pore and continuum scales, surface tension dominates over gas inertia. At the same injection rate, inertial forces exceed viscous forces up to roughly 50 mm from the inlet, but this range is still well below the size of the sandbox (125 mm to each side), and capillary forces remain the controlling factor throughout. Overall, these estimates show that inertial forces do not determine the displacement patterns observed in our experiments; capillary and viscous forces do.”

4. Conclusions:
Please restructure the conclusion as follows:
Paragraph 1, Overall Summary: Begins with a broad overview of the topic, outlining the scope, key themes, and the progression of the review. Sets the context by connecting existing knowledge with emerging challenges or gaps.
Paragraph 2, Thematic or Point-by-Point Breakdown: Followed by a structured list of conclusions or insights, usually numbered or bulleted. Each point presents a distinct observation, finding, or implication, often supported by comparisons or qualifications. The list moves logically from theoretical foundations to experimental methods and finally to practical or applied considerations.
Paragraph 3, Final Integrative Conclusion: Ends with a synthesis or reflection, often forward-looking. Highlights the practical relevance, implications for future work, or broader significance of the findings.

Ans: We thank the reviewer for the suggestion. After consulting established guidance on scientific writing and surveying relevant literature, we confirm that the conclusion includes the essential components expected of this section. We have, however, refined its structure to improve clarity and coherence. Nonetheless, we respectfully refrain from reorganizing it into exactly three paragraphs, as this constraint does not further enhance the presentation of our findings. Modified Conclusions section:

“In this study, we compared the performance of four macroscopic IP models against the data from nine experiments. The experiments featured gas injections in homogeneous water-saturated sand. For comparison, we used time-matching and (Diffused) Jaccard coefficient(s). For the first time, these models are tested in transitional and continuous gas-flow regimes. We identified the strengths and weaknesses of these modelling strategies for simulating gas flow in water-saturated sand. We also calibrated a few key parameters of these models.

Concerning the compared models, we conclude that Models 1 and 2 should not be used for the transitional and continuous regimes of gas flow discussed in this study. In particular, these models are completely weak for experiments at higher injection rates. In previous studies, IP models have been used extensively only in the capillary flow domain. Our results show that IP models at a macroscopic scale with variation as Model 3 can be used in the transitional gas flow regime but are unfit for use in the continuous gas flow regime. In their present state, Models 3 and 4 can be used with blurring for large-scale applications in the continuous gas flow regime, where the details of the gas-cluster structure are insignificant. Thus, the exact use depends on the specific application. Models 3 and 4 perform better because they can partially account for the viscous effects found at high gas injection rates.

Although, Model 3 and Model 4 show some promise in performance, further research towards refining their rules for gas-invasion, water-re-invasion, finger branching, and so on needs to be done. A possible extension could be a mix of Model~3's rule of invading more blocks per step combined with a stochastic invasion rule similar to that of Model~4. The rule for this extension would also need to be adapted to better mimic the gas flow behaviour in the continuous flow regime, e.g., with finger invasion rules enabling the growth of multiple parallel thick fingers.

Regarding how to judge model-experiment agreement, we emphasize the importance of comparing results at an appropriate level of detail. In this context, blurring of images can be used as an efficient tool to reduce the detailed level of information in the images, depending on the application and the scale of interest. It is pointless to ask for a pixel-to-pixel match at and above the scale of the experiments used in this study, given the strong dependence of gas flow on pore-scale aspects of the porous medium (here: sand pack). This exercise can thus help justify the use of models like 3 or 4 for such applications.

A primary conclusion from our investigation of the sensitivity of the model parameters is that the underlying structure of the $T_e$ fields is a critical input for these models. Moreover, the best models (3 or 4) are also the most sensitive to this input. Further research could be conducted to identify the underlying structure of the $T_e$ fields, e.g. using geostatistical inversion methods.

Overall, our presented framework allowed us to compare several competing macroscopic IP models based on highly resolved image data. The employed Jaccard metric and its adjustable blurring parameter enabled an objective comparison that can focus on relevant scales of comparison and allows to fade out unnecessary pixel-wise detail. And finally, it provided relevant and valuable insights for further model development.”

## Response to Reviewer #2

**Reviewer #2:** This manuscript presents a comparative framework for evaluating four macroscopic invasion percolation (IP) models against experimental data from gas injection in water-saturated sand across transitional and continuous flow regimes. The authors use a diffused Jaccard coefficient approach to rank model performance and identify that Models 3 and 4 (which incorporate rules for multiple block invasion and stochastic selection, respectively) outperform traditional IP models in these previously unexplored flow regimes.

- Novel Evaluation Framework: The introduction of the diffused Jaccard coefficient and a robust, image-based comparison method provides a significant advancement for model-data comparison in porous media research.

- Clear Problem Definition: The manuscript addresses a well-defined gap in the literature regarding the suitability of IP models for flow regimes beyond traditional discontinuous cases.

- Comprehensive Experimental Basis: Nine well-characterized experimental datasets, covering triplicates at different flow rates, offer a strong basis for benchmarking.

Major comments:

- The abstract is overly technical and method-focused. It under-emphasizes the broader significance of the work.
Revise to highlight (a) the problem addressed, (b) the novelty of the framework, (c) the key results (e.g., Model 3 generally outperforms others), and (d) implications for practical applications such as CO₂ storage or groundwater protection.

Ans:

We thank the reviewer for this recommendation. We have modified the abstract by addressing the points mentioned in the comment as follows:

“Predicting gas migration in water-saturated porous media (a multiphase flow problem) is essential for applications such as carbon dioxide sequestration, hydrogen storage, and groundwater remediation. This process is challenging because gas migration can occur either as continuous or discontinuous flow, depending on the flow rate and the properties of the porous medium. Numerous variations of Invasion-Percolation (IP) models can simulate multiphase flow across different scales (from pore-scale to macroscopic), but the literature suggests that IP models are best suited for the discontinuous gas flow regime; other regimes have not been systematically evaluated. Here, we present a quantitative framework for comparing and ranking macroscopic IP models against high-resolution experimental data from transitional and continuous gas flow regimes. The analysis employs a diffused Jaccard coefficient to measure the similarity between model outputs and time-series two-dimensional images from gas injection experiments in water-saturated sand. To represent pore-scale heterogeneity, we run each model version on several random realizations of the initial entry-pressure field. The Jaccard coefficient was averaged over all realizations per model version to evaluate performance and calibrate model parameters. Depending on the application domain, some macroscopic IP model versions are suitable for these previously unexplored flow regimes. We also find that heterogeneity in the initial entry-pressure field strongly influences model performance. The observed applicability of IP models in these regimes can substantially reduce computational costs compared to continuum-based models for applications such as carbon dioxide storage and groundwater protection, although their suitability ultimately depends on the specific research question being addressed. The proposed comparison framework is not limited to gas–water systems in porous media but generalizes to any modelling situation accompanied by spatially and temporally resolved data.”

We hope that the reworked abstract is to your liking, and that the additional details provided on the model-wise conclusions—kept deliberately concise—are sufficient. Since the primary focus of this study is the comparison methodology, we aimed to keep the abstract centered on this aspect and to avoid overloading it with model-specific details that would detract from the main contribution.

- Limited Physical Understanding: The manuscript lacks sufficient mechanistic insight into why certain models perform better. The authors acknowledge that Models 3 and 4 can "partially consider viscous effects" but do not provide clear physical justification for how multiple block invasion or stochastic selection actually represents viscous processes.

Ans: We thank the reviewer for this comment. In the experiments, a thickening of the gas channels is observed as gas injection rate increase. As injection rate increases, capillary-pressure-driven randomness in the invasion pattern becomes less important than viscous forces. Thus, the gas phase becomes more connected producing thicker fingers. We have emphasized the physical justification for how multiple block invasion or stochastic selection actually represents viscous effects in the revised manuscript as follows:

In lines 373-377:

“Consequently, instead of invading only the block with the lowest entry threshold, the $nb$ lowest-threshold candidates are selected. This reduces the controlling influence of $T_e$ and thereby reflects a shift from capillary-dominated behaviour towards a regime in which viscous effects become more prominent, as is characteristic of gas flow at higher injection rates.”

In lines 396-401

“The distinction between Model 3 and Model 4 is that Model 3 weakens the strict control of T_e by advancing several blocks at each step, whereas Model 4 does so by selecting a single block stochastically. In both cases, relaxing the strict ordering of thresholds shifts the invasion pattern away from a purely capillary-dominated process and towards behaviour in which viscous effects play a stronger role, as is expected at higher gas-injection rates.”

Furthermore, Models 3 and 4 are well established and thoroughly documented in the cited literature. In this work, we have tested these existing modelling strategies.

-The manuscript claims that the framework is generalizable beyond gas-water systems, but this is not strongly substantiated. Provide concrete examples of how this framework could be applied to other multiphase systems (e.g., oil-water, CO₂ sequestration) or even to data-driven models.

Ans:

We thank the reviewer for this comment. We have added the following text in lines 225-234 in the manuscript to provide examples where our approach may be extended.

“Furthermore, the model comparison framework developed in this study is not limited to the four models tested here; it is a versatile tool that can also be applied to other models. For example, in multiphase flow problems in porous media, this framework can be applied to systems such as oil recovery and carbon dioxide sequestration (with pore network modeling, micro-CT, or capillary pressure experiments) \cite{Wang2023}. It can also be extended to evaluate data-driven models in fields such as medical tomography \cite{Rainio2024} and digital rock physics \cite{Reinhardt2022}. Our work demonstrates the utility and robustness of this framework, showcasing its potential for broader application in the analysis and improvement of numerical models that predict highly resolved observable data. ”

- Address Model Limitations: Explicitly discuss the physical meaning of unrealistic gas saturation values and their implications for model applicability.

Ans: We would like to thank the reviewer for this comment. This point is already addressed in the manuscript (see lines 767–793), where we explain the non-physical gas saturation values and their implications for model applicability. We have added a brief clarification to make this more visible.

“Thus, the value of S_g=0.12 for the entire gas cluster is substantially lower than that observed and reported in \cite{VanDeVen2020}, **which implies that the model overestimates the overall extent of the gas distribution.**”

- Expand Experimental Scope: Include additional porous media types or acknowledge this as a significant limitation.

Ans:

We thank the reviewer for this constructive suggestion. While incorporating additional porous media types would indeed broaden the experimental scope, this extension is beyond the objectives of the present study. We have now explicitly acknowledged this as a limitation in the revised manuscript in lines 296-299

“We use experimental datasets that involve one type of porous medium; however, this does not influence the validity or structure of the proposed comparison framework. Variations in porous media can affect individual model performance, but not the overall applicability of the model comparison framework.”

Minor comments:
- The manuscript is generally well-written, though some sections (especially methodological details) could be condensed or clarified for broader accessibility.

Ans: We critically looked at the entire manuscript for possible places to condense, or to clarify. Clarification and condensation are somewhat competing. Therefore, we followed the more detailed comments of the two reviewers to guide this process and are confident that the manuscript is now in an excellent shape.

- Extend analysis to 3D models or justify 2D limitation

Ans: We would like to thank the reviewer for this suggestion. However, we would like to point out that the experiments were quasi 2D, and hence the choice of 2D models. Moreover, Mumford et al. (2015) demonstrated that 2D and 3D representations of similar experiments yield comparable invasion behavior, with 3D models primarily affecting thickness-averaged gas saturation rather than the underlying mechanisms. For these reasons, we would not be extending the analysis to 3D models.

- Systematic evaluation of parameter sensitivity

Ans:

We thank the reviewer for this thoughtful comment. We have investigated the sensitivity of key parameters, specifically the entry-threshold fields and gas saturation values, within our framework. These parameters are the most influential in the current modeling setup, and therefore additional parameter testing was not deemed necessary.

- Consider additional comparison metrics beyond Jaccard coefficient

 Ans:

We thank the reviewer for this valuable comment. We would like to clarify that we had previously analyzed several alternative metrics (e.g., Earth Mover’s Distance) before selecting the Jaccard coefficient for this framework. The selection was based on its simplicity, computational efficiency, and suitability for binary image data. Hence, we continue to use the (Diffused) Jaccard coefficient in this study as also in our previous study (Banerjee et. al. 2020) and do not include additional metrics in the manuscript.

## Closing Remarks

We greatly appreciate the time and effort that you and the reviewers have invested in providing feedback. We hope that the revised manuscript meets your expectations and look forward to your favorable consideration.
Sincerely,
Dr.-Ing. Ishani Banerjee

**References**

1. Van De Ven CJC, Mumford KG. Characterization of Gas Injection Flow Patterns Subject to Gravity and Viscous Forces. Vadose Zone Journal 2019;18(1):1–11. doi:10.2136/vzj2019.02.0014.
2. Mumford KG, Hegele PR, Vandenberg GP. Comparison of Two-Dimensional and Three-Dimensional Macroscopic Invasion Percolation Simulations with Laboratory Experiments of Gas Bubble Flow in Homogeneous Sands. Vadose Zone Journal. 2015;14(11):0. doi:10.2136/vzj2015.02.0028.
3. Selker JS, Niemet M, Mcduffie NG, Gorelick SM, Parlange JY. The Local Geometry of Gas Injection into Saturated Homogeneous Porous Media. Transp Porous Med. 2006;68:107–127. doi:10.1007/s11242-006-0005-0.
4. Banerjee I, Guthke A, Van De Ven CJC, Mumford KG, Nowak W. Overcoming the model-data-fit problem in porous media: A quantitative method to compare invasion-percolation models to high-resolution data. Water Resources Research. 2021;57. doi:10.1029/2021WR029986.
