# Supplementary material for: A framework for objectively comparing competing invasion percolation models based on highly-resolved image data
Source: PLoS One. 2026 Mar 23;21(3):e0327414. doi: 10.1371/journal.pone.0327414 (PMC13008257; doi:10.1371/journal.pone.0327414)
Supplement: Attachment — Submitted filename: Response to Reviewers_Rev2.docx [file pone.0327414.s017.docx]

Response to Reviewers:

Dear Prof. Reuben O'Dea

We would like to sincerely thank you and the reviewer 1 for your constructive comment on our manuscript: PONE-D-25-31748: “A framework for objectively comparing competing invasion percolation models based on highly-resolved image data”. We have carefully revised the manuscript accordingly. Below, we provide a detailed response to the comment, along with a summary of the changes made in the revised version.

Response to Reviewer 1:

Reviewer #1: The authors have mainly addressed the comments from the last round of review. I have no additional comments on it except for one raised by the other reviewer (Reviewer #2).
The authors answered that heterogeneity in the initial entry-pressure field strongly influences model performance (Lines 47-48). You answered this well in your response letter and included additional information in the abstract. However, you forgot to mention this part in the main text and only put minor information in Line 742 (the uncertainty of the geological heterogeneity in their experimental setup). Could you please add the essential findings and discussion not only in the abstract and response letter but also in the main text, in the results and discussion chapters?
Also, another good study investigated the role of local heterogeneity in the initial entry pressure and scale effects in the capillarity-saturation relationship, which may help to support this finding (“the initial entry-pressure field strongly influences model performance”).
Yan, G., Bore, T., Schlaeger, S., Scheuermann, A., & Li, L. (2022). Investigating scale effects in soil water retention curve via spatial time domain reflectometry. Journal of Hydrology, 612, 128238.

Ans: Thank you for your question, which prompted us to re-examine the terminology used in the manuscript. In doing so, we realized that our parallel use of *entry pressure fields* and *invasion threshold fields* could lead to confusion, even though this was not explicitly raised.

To clarify this point, we now consistently use the term **invasion threshold fields** throughout the abstract and main text. At the scale of our model, the invasion threshold represents an effective quantity that combines the local entry pressure field with the hydrostatic pressure field.

Further, the detailed analyses describing how model performance depends on the invasion threshold fields were already presented in Sections **4.1.1** and **4.1.2**. To improve readability and better guide the reader, we have now explicitly synthesized these existing results and added a short summary in **Section 4.2**, highlighting the central role of invasion threshold uncertainty in model performance.

“The analysis across 500 $T_e$ realisations in Section 4.1.2 demonstrates that model performance is highly conditional on the invasion threshold field. Deterministic model variants perform exceptionally well only for a narrow subset of favourable $T_e$ fields, while more flexible formulations show greater robustness when the $T_e$ field poorly represents the experimental medium. This sensitivity confirms that $T_e$ is a critical input for the models.”

We thank the reviewer for the helpful suggestion of the study: “*Yan, G., Bore, T., Schlaeger, S., Scheuermann, A., & Li, L. (2022). Investigating scale effects in soil water retention curve via spatial time domain reflectometry. Journal of Hydrology, 612, 128238*”. However, while experimental measurements may indeed be subject to such uncertainties, our discussion is framed from the perspective of model-concept uncertainty, focusing on how model performance is affected when the true invasion threshold field cannot be represented, rather than on experimental measurement approaches, and we therefore do not cite the study.

We hope that the revised manuscript meets your expectations and look forward to your favorable consideration.

Sincerely,

Dr.-Ing. Ishani Banerjee
